# TC3-VLM: A Vision–Language Model for Tactical Combat Casualty Care

**DOI:** 10.1109/access.2026.3726225

**Published:** 2026-08-24

**Authors:** JUNSEOB KIM, JADE CHNG, AYMAN ALI, VICTOR MOAS, SUNIL HWANG, RISHIKESAN KAMALESWARAN

**Affiliations:** 1 Department of Electrical and Computer Engineering, Duke University, Durham, NC 27708, USA; 2 Department of Biomedical Engineering, Duke University, Durham, NC 27708, USA; 3 Department of Surgery, Duke University, Durham, NC 27708, USA; 4 Department of Mathematics, Korea Military Academy, Seoul 01805, South Korea; 5 Department of Anesthesiology, Duke University, Durham, NC 27708, USA

**Keywords:** Clinical decision support, low-rank adaptation, medical video understanding, Tactical Combat Casualty Care, vision–language model

## Abstract

Tactical Combat Casualty Care (TC3) defines the current standard of military trauma care in the pre-hospital and battlefield settings, where life-saving decisions are made while operating at the extremes of human performance, under high stress, and with limited information. Despite growing interest in AI-assisted battlefield medicine, visual reasoning remains underexplored in existing systems. Recent advances in vision–language models (VLMs) enable the integration of visual information with medical reasoning, but their application to combat casualty care remains limited. To address this gap, we present TC3-VLM, the first VLM explicitly tailored for combat casualty care. Using a dual question–answer (Q&A) generation framework, we create visual-grounded Q&A to capture spatial and situational reasoning alongside caption-based Q&A to encode protocol knowledge, resulting in a domain-specific dataset of 7,585 Q&A pairs derived from over 52 hours of TC3 videos. We then fine-tune multiple VLM backbones via Low-Rank Adaptation across varying model scales. Experiments demonstrate that fine-tuned models consistently outperform their baseline counterparts, with TC3-VLM surpassing a 72B-parameter general-purpose VLM despite being significantly smaller in scale. Ablation and a TC3-grounded error analysis indicate that visual grounding is the primary driver of these gains and that fine-tuning reduces safety-relevant errors, positioning TC3-VLM as a training and reference aid for combat casualty care.

## I. INTRODUCTION

In battlefield trauma, survival is often decided within minutes, and most preventable deaths trace to a small set of causes such as uncontrolled hemorrhage [1], [2], [3]. Tactical Combat Casualty Care (TC3) is the standardized framework developed to manage these injuries in prehospital and battlefield environments, where care proceeds under severe resource constraints, limited situational awareness, and restricted access to advanced medical support [4], [5]. At the point of injury, responders must identify wounds, recognize medical equipment, locate anatomical landmarks, assess intervention placement, and monitor procedural execution, often before any physiological measurement is available [6]. Combat casualty care is therefore, at its core, a visual and spatial decision-making task that depends on accurate scene interpretation and timely intervention under uncertainty.

The demands of casualty care are intensifying from two directions at once. In contested environments, rapid evacuation can no longer be assumed, and casualties may require prolonged field care for hours or even days before reaching definitive treatment [7], [8]. At the same time, the life-saving skills such care depends on deteriorate within months of training, particularly among providers who perform these interventions infrequently [9]. As care durations lengthen and provider proficiency declines, the need grows for tools that can reinforce training and support treatment decisions at the point of injury, where responders often have little to rely on beyond the resources immediately at hand.

Despite growing interest in artificial intelligence (AI) for military and prehospital medicine, existing approaches address only part of this requirement. Most work has focused on physiological monitoring and prediction, including shock and hemorrhage detection from vital-sign time series [10], [11], reinforcement-learning approaches for fluid and resource allocation [12], and broader autonomy and monitoring frameworks [13], [14], [15], [16]. Reviews of prehospital-trauma AI similarly conclude that the field remains dominated by tabular prediction tasks [17]. Although these systems provide valuable physiological decision support, they largely ignore the visual interpretation of the injury scene—the information source most immediately available to a responder.

Vision–language models (VLMs) offer a natural foundation for addressing this missing visual component and have demonstrated strong multimodal reasoning performance across a range of medical applications [18], [19], [20], [21]. However, existing medical VLMs are developed primarily for hospital-centered imaging and biomedical workflows rather than combat casualty care. Consequently, they are not trained to reason within the procedural priorities, treatment constraints, and intervention guidelines that govern TC3. Because civilian and TC3 protocols diverge in several high-consequence situations, models trained on general medical practice may generate recommendations that conflict with TC3 doctrine or fail to account for battlefield constraints. Effective adaptation to this domain therefore requires more than multimodal medical knowledge; it requires explicit alignment with TC3 doctrine.

To address this gap, we present TC3-VLM, the first vision–language model specialized for Tactical Combat Casualty Care. We construct a domain-specific dataset of 7,585 question–answer (Q&A) pairs derived from 374 publicly available TC3 instructional and field videos comprising more than 52 hours of footage. The dataset is generated through two complementary supervision pipelines: a visual-grounded branch that uses a large VLM to generate scene-based supervision from sampled video frames, capturing spatial and procedural reasoning, and a caption-based branch that uses a large language model (LLM) to generate protocol-oriented supervision from refined automatic speech recognition transcripts. Using this dataset, we adapt Qwen-VL backbones (2B, 7B, and 8B parameters) through Low-Rank Adaptation (LoRA). We evaluate TC3-VLM using quantitative benchmarks, ablation studies, and TC3-grounded safety analyses to assess the impact of domain-specific adaptation on combat casualty care tasks.

The novelty of this work lies in constructing a TC3-specific multimodal training resource and adapting general-purpose VLMs to this data-scarce, safety-critical domain. Our contributions are summarized as follows:

- **The first TC3-specialized vision–language model.** We present TC3-VLM, the first vision–language model built for TC3. To overcome the absence of any TC3 training resource, we construct a pipeline that converts instructional combat-medicine video into multimodal procedural supervision and use it to specialize TC3-VLM.
- **Domain specialization outweighs scale.** Our 8B model surpasses a 72B general-purpose baseline on TC3 reasoning. An ablation across visual-grounded and transcript-derived supervision shows that the two sources play complementary roles in this gain.
- **A TC3-grounded safety-error analysis.** We identify safety-relevant failure modes of general-purpose models and show that TC3-specific adaptation substantially reduces these errors.
- **A comprehensive evaluation framework for TC3 VLMs.** We evaluate TC3-VLM with complementary automatic and model-based metrics, and report where current evaluation methods succeed and where they fall short.

The remainder of this paper is organized as follows. Section II reviews related work in medical and multimodal VLMs and battlefield AI. Section III describes the dataset, the dual Q&A generation pipeline, and the LoRA adaptation method. Section IV reports the experimental setup, main results, ablations, and error and safety analysis. Section V discusses limitations and future work, and Section VI concludes.

## II. RELATED WORK

Table 1 situates TC3-VLM against representative prior work spanning medical VLMs, medical question answering, and surgical or medical video understanding.

### A. MEDICAL AND MULTIMODAL VISION–LANGUAGE MODELS

Medical visual question answering (Med-VQA) and medical VLMs have grown rapidly. Clinician-authored radiology benchmarks such as VQA-RAD [24] and the knowledge-enhanced SLAKE [30] pair questions with single diagnostic images, and PathVQA [25] scales pathology VQA by a semi-automated caption-to-QA pipeline over textbook figures. Instruction-tuned generative systems follow the same image-centric premise: LLaVA-Med [22] tunes a vision–language assistant on PubMed Central figure–caption pairs with model-generated instructions, PMC-VQA [23] casts Med-VQA as open generation over 227K image–question pairs, and Med-PaLM 2 [18] reaches expert-level accuracy on text-based medical examinations. Recent large-scale evaluations further reveal that medical-specialized VLMs do not consistently outperform larger general-purpose models [31], highlighting the need to better understand the conditions under which domain specialization is beneficial. Despite these advances, existing medical VLM benchmarks remain focused on diagnostic reasoning from static images and do not address procedural decision-making in resource-constrained trauma environments. Moreover, current datasets provide little supervision aligned with TC3, where treatment decisions are shaped by battlefield constraints, limited resources, and doctrine-specific intervention priorities.

### B. LEARNING MEDICINE FROM VIDEO

Recent work has extended medical vision–language learning beyond static images to instructional and procedural video. MedVidQA [26] provides human-annotated question answering over medical instructional videos, and Open-BiomedVid [27] fine-tunes a Qwen-VL backbone using question–answer pairs derived from public educational biomedical videos. In the surgical setting, LLaVA-Surg [28] builds a multimodal surgical assistant through LLM-driven two-stage QA generation from public lecture videos. A longer recognition-oriented tradition, including phase and tool recognition with EndoNet [32], transformer-based surgical VQA [29], and egocentric open-surgery phase recognition [33], learns from curated operating-room video under task-specific or closed-label taxonomies. Although these studies show the value of video for medical AI, they primarily address general biomedical education, surgical assistance, or operating-room recognition. They do not target point-of-injury trauma care, where visual cues must be interpreted under resource constraints, heterogeneous field environments, and TC3-specific treatment priorities.

### C. BATTLEFIELD AND PREHOSPITAL MEDICAL AI

AI for combat and prehospital trauma care has so far been developed primarily around non-visual inputs and predictive decision support. TC3-specific studies include machine-learning shock detection from vital-sign time series [10], externally validated hemorrhage- and shock-risk prediction [11], reinforcement learning for casualty-care resource allocation [12], and broader roadmaps for autonomy, digital twins, and wearable-biosensor early warning on the future battlefield [13], [14], [15], [16]. Similarly, a scoping review of prehospital-trauma AI shows that existing work is dominated by tabular predictive tasks, including life-saving-intervention prediction, triage, and survival estimation [17]. These studies provide important foundations for trauma-care decision support, but they do not address open-ended visual reasoning over casualty-care procedures or doctrine-grounded instruction from multimodal observations. This gap is particularly important because emergency-care models trained or prompted in general clinical contexts may not reliably capture the priorities and constraints of TC3. For example, general-purpose large models have been reported to over-triage and under-detect high-acuity patients in emergency settings [34], which motivates domain-grounded evaluation and adaptation in high-stakes trauma scenarios.

### D. EVALUATING GENERATIVE MEDICAL MODELS

Evaluating generative medical models remains challenging, particularly when standardized benchmarks are unavailable or when outputs require expert judgment. LLM-as-Judge protocols offer a scalable alternative and have been shown to align with human preferences, but they are also known to exhibit position, verbosity, and self-enhancement biases [35], [36]. Chain-of-thought-based evaluators such as G-Eval [37] can further favor LLM-generated text, and recent work shows that LLM evaluators may recognize and preferentially score their own generations [38]. These concerns are especially relevant for domain-specific medical generation, where training data, candidate outputs, and judge models may come from overlapping model families. Separately, retrieval-augmented generation systems such as MMed-RAG [39] and Almanac [40] represent an alternative approach to improving factual grounding and safety without updating model weights.

### E. PARAMETER-EFFICIENT DOMAIN ADAPTATION

Parameter-efficient fine-tuning provides a practical approach to domain specialization when curated training data are limited. LoRA [41] introduces trainable low-rank updates into pretrained models while keeping the original backbone largely frozen, reducing the computational cost of adaptation while preserving pretrained representations. This paradigm has become especially relevant for adapting large VLM backbones, including the Qwen-VL family [42], [43], [44], to specialized domains. For TC3, where curated multimodal data are scarce relative to large biomedical corpora, parameter-efficient adaptation offers a feasible route to study domain specialization without the cost of full-model retraining.

## III. METHOD

**FIGURE 1.**
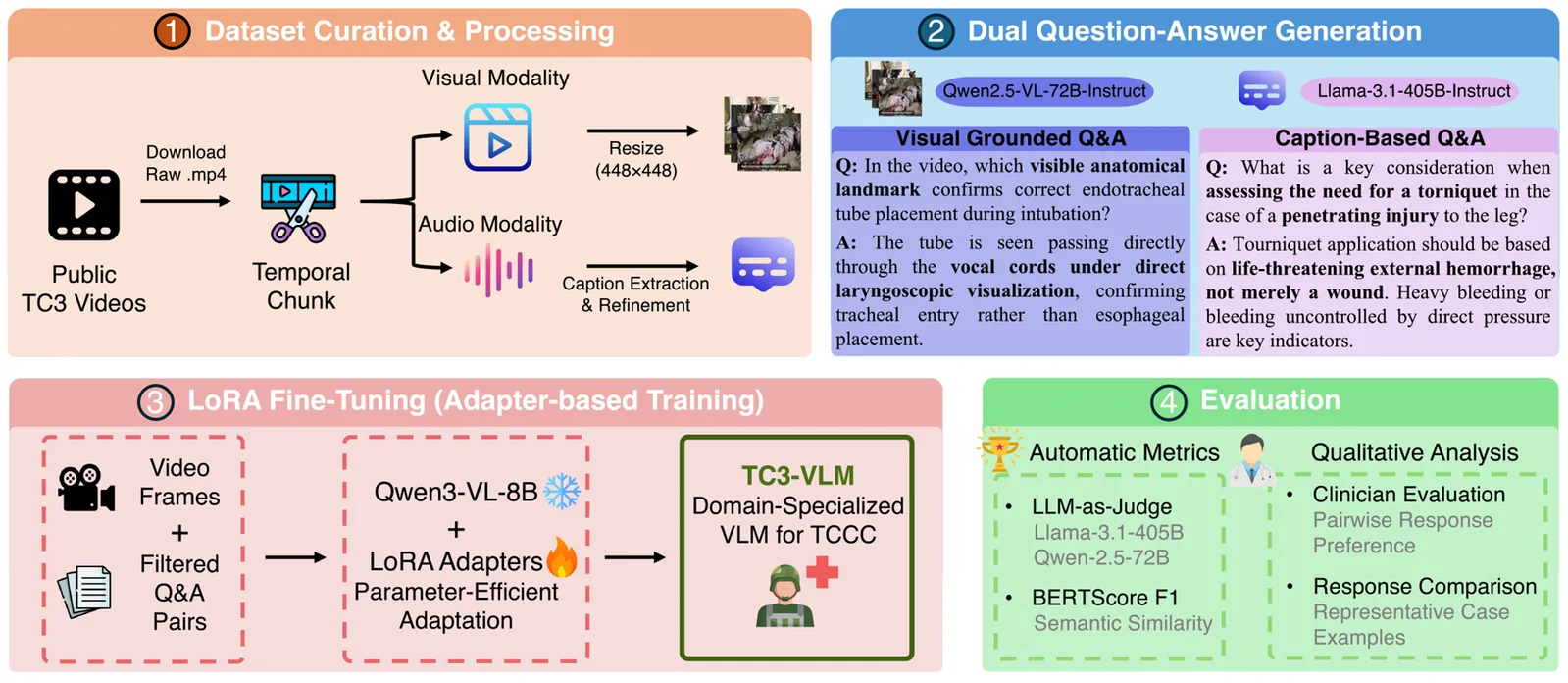
Overview of the TC3-VLM framework. (1) Public TC3 videos are temporally chunked, with visual frames and audio transcripts processed separately to extract structured supervision signals. (2) A dual Q&A generation pipeline generates visually grounded supervision for spatial and situational understanding and caption-based supervision for procedural reasoning. (3) The generated Q&A pairs are used to fine-tune a pretrained Qwen3-VL-8B model via LoRA-based domain adaptation. (4) The resulting TC3-VLM is evaluated through automatic metrics alongside clinician assessment.

Figure 1 illustrates the overall architecture and training pipeline. In this section, we describe each component in detail.

### A. DATASET CURATION AND PROCESSING

#### 1) CURATION STRATEGY

To construct a dataset tailored to TC3, we defined structured search queries covering key components of the TC3 guidelines [1]. Videos were collected from publicly accessible sources, including YouTube and TC3-related educational websites. To capture both protocol-level instruction and real-world procedural execution, the dataset includes formal educational materials as well as field treatment footage. The initial search identified 476 candidate videos, which were filtered to remove irrelevant or non-downloadable content. The final dataset consists of 374 videos with a total duration of approximately 52.5 hours, spanning diverse TC3 procedures and operational environments.

#### 2) VIDEO PREPROCESSING

The collected videos were segmented into fixed-duration chunks to accommodate long-form content within manageable input lengths for VLM training while maintaining procedural coherence. Videos exceeding 8 minutes were split into 6-minute segments with 2-minute overlaps to preserve procedural continuity across segment boundaries. This process produced a total of 829 chunks with a mean duration of 292.6 seconds. To standardize heterogeneous input resolutions, all frames were resized to 448 × 448 pixels, aligning with the 28 × 28 pixel-per-token encoding scheme used in Qwen-VL models [42], [44].

#### 3) CAPTION EXTRACTION AND REFINEMENT

Captions were extracted from the audio tracks using platform-provided subtitles when available and otherwise transcribed using OpenAI Whisper [45]. To improve clarity and medical specificity, raw captions were refined using Qwen2-7B-Instruct [46]. The model was prompted to remove filler words and noise markers, normalize medical terminology, and produce a concise multi-sentence description focused on procedural steps and key clinical actions. Table 2 shows representative raw and refined captions.

### B. DUAL QUESTION–ANSWER GENERATION

To systematically investigate supervision across these domains, we construct a dual Q&A dataset consisting of caption-based and visual-grounded subsets.

#### 1) CAPTION-BASED Q&A

Caption-based Q&A pairs capture procedural, clinical, and tactical knowledge conveyed through audio narration. We prompted Meta-Llama-3.1-405B-Instruct [47] to generate question–answer pairs directly from the refined captions, ensuring that responses were grounded exclusively in transcript-derived content. Prompts encouraged diverse reasoning types, including procedural sequencing, protocol application, equipment usage, timing considerations, and tactical decision-making. This subset provides structured textual supervision aligned with formal TC3 instruction.

#### 2) VISUAL-GROUNDED Q&A

Many critical TC3 competencies—such as anatomical landmark identification, instrument positioning, hand placement, and spatial verification of procedural execution—are not fully captured by transcript-derived supervision. To address this limitation, we employed Qwen2.5-VL-72B [43] to analyze sampled video frames and generate Q&A pairs grounded in direct visual evidence. Prompt designs emphasized questions that require explicit visual cues, thereby providing supervision complementary to the caption-based component.

#### 3) QUALITY FILTERING

All generated Q&A pairs were evaluated using an LLM as an automated judge [47]. Each pair was scored on *factual correctness*, *completeness*, *clinical relevance*, *specificity*, and *safety* (1–5 scale). Q&A pairs with an average score below 3 were discarded to ensure minimum quality and safety standards. After filtering, the final dataset comprises 7,585 Q&A pairs. Representative examples are shown in Figure 1.

### C. DOMAIN-ADAPTIVE FINE-TUNING

We perform domain-adaptive fine-tuning of Qwen-VL models for TC3 using LoRA [41]. Instead of updating all parameters, LoRA introduces trainable low-rank adapters while freezing the pretrained backbone, enabling efficient domain specialization and preserving the visual–language representations learned during pretraining. This approach is well suited to our setting, where domain-specific training data is limited relative to large-scale medical corpora.

Let $\theta_{0}$ denote the frozen backbone parameters and Δθ={A,B} the trainable adapter parameters, such that $\theta =\theta_{0}+\Delta \theta$. For each target weight matrix $W\in R^{d\times k}$, the adapted weights are parameterized as:

(1) $$W^{\prime }=W+\frac{\alpha }{r}BA$$

where $B\in R^{d\times r}$ and $A\in R^{r\times k}$ are trainable low-rank matrices, and *r* and *α* control the rank and scaling of the adaptation. While the ratio α/r regulates the magnitude of the injected weight update, the rank *r* determines the intrinsic dimensionality of the weight update, controlling the number of learnable directions in parameter space and thus the adapter’s expressive capacity. Adapters are applied to all attention and feed-forward projection layers in both the visual encoder and language backbone, ensuring adaptation of both perceptual and reasoning components to the TC3 domain.

The model is trained using a causal language modeling objective to autoregressively generate answer tokens conditioned on visual tokens **v** and question tokens **x**:

(2) $$ℒ=-\overset{T}{\underset{t=1}{\sum }}\text{log}\;P_{\theta }\left(y_{t}|v,x,y_{<t}\right)$$

where *y*_1:*T*_ denotes the answer sequence. Supervision is applied only to answer tokens by masking question and padding positions, thereby focusing optimization on clinically grounded response generation rather than input reconstruction.

## IV. EXPERIMENTS AND RESULTS

We use the dataset described in Section III, partitioned at the video level (261/56/57 videos; approximately 70%/15%/15%) into training, validation, and test splits to prevent leakage, yielding 5,409, 975, and 1,201 Q&A pairs, respectively. We fine-tune three backbones (Qwen2-VL-2B, Qwen2-VL-7B, and Qwen3-VL-8B-Instruct) under a unified LoRA training protocol, ablating five configurations with varying *r* and *α* as well as three training data composition modes (caption-based, visual-grounded, and combined). Because TC3 has no standardized generative benchmark, we evaluate models using complementary metric families: judge-independent reference-overlap metrics, including BERTScore F1 [48] and ROUGE-L [49], and an LLM-as-Judge protocol with two independent judges following Med-PaLM 2’s evaluation framework [18], adapted for the TC3 domain. Each answer is scored on five 1–5 axes—medical accuracy, protocol adherence, completeness, actionability, and safety—averaged into an overall score. This design reduces dependence on any single evaluator and allows discrepancies between judge-based and reference-based metrics to be analyzed explicitly.

### A. IMPLEMENTATION DETAILS

All three backbones are fine-tuned identically. LoRA adapters are applied to the attention (*q*, *k*, *v*, *o*) and feed-forward (gate, up, down) projections with dropout 0.05 and no bias terms, and we sweep five rank/scaling settings (*r*, *α*) ∈ {(8, 8), (16, 16), (32, 32), (32, 64), (16, 64)}. Each model is trained for three epochs with the AdamW optimizer, a learning rate of 1×10^−4^ on a cosine schedule with warmup, and weight decay 0.01; a per-device batch size of one with gradient accumulation of eight across eight NVIDIA H200 GPUs yields an effective batch size of 64. Each clip is represented by up to 28 sampled frames within a 12,288-token context.

### B. MAIN RESULTS

Table 3 presents evaluation results across model scales. Fine-tuning consistently improves performance across all evaluated dimensions, indicating that domain-specific adaptation remains effective at different model sizes. Qwen3-VL-8B achieves an overall gain of +0.557 (Cohen’s *d_z_* = 0.51, 95% CI [+0.49, +0.62]), while Qwen2-VL-7B and Qwen2-VL-2B gain +0.757 (*d_z_* = 0.67, [+0.69, +0.82]) and +0.416 (*d_z_* = 0.34, [+0.35, +0.49]), respectively. Across the five scoring axes, per-axis effect sizes range from *d_z_* = 0.28 to 0.80, spanning small to large effects. Among all configurations, TC3-VLM (Qwen3-VL-8B, *r*=16, *α*=64, visual-grounded) achieves the highest overall LLM-Judge score of 4.584, surpassing the Qwen2.5-VL-72B baseline despite its smaller parameter count. The most pronounced gains are observed in Completeness and Actionability, suggesting that visual-grounded training provides procedural context that enhances both the completeness of clinical detail and the actionability of field-executable guidance. BERTScore improvements further corroborate these findings through an independent semantic similarity metric.

To validate our LLM-as-Judge protocol, we analyze inter-judge agreement between Llama-3.1-405B and Qwen2.5-72B across model scales and settings (Table 4). Both Pearson *r* and Spearman *ρ*, reflecting linear and rank-order agreement respectively, exceed 0.7 in the majority of configurations. Additionally, over 75% of responses receive scores within one point between the two judges, and low mean absolute error (MAE) values further support close score-level agreement [50]. These results indicate that our evaluation framework yields consistent and reproducible assessments of TC3-specific medical reasoning while mitigating the influence of individual judge variability.

### C. ABLATION AND QUALITATIVE ANALYSIS

**FIGURE 2.**
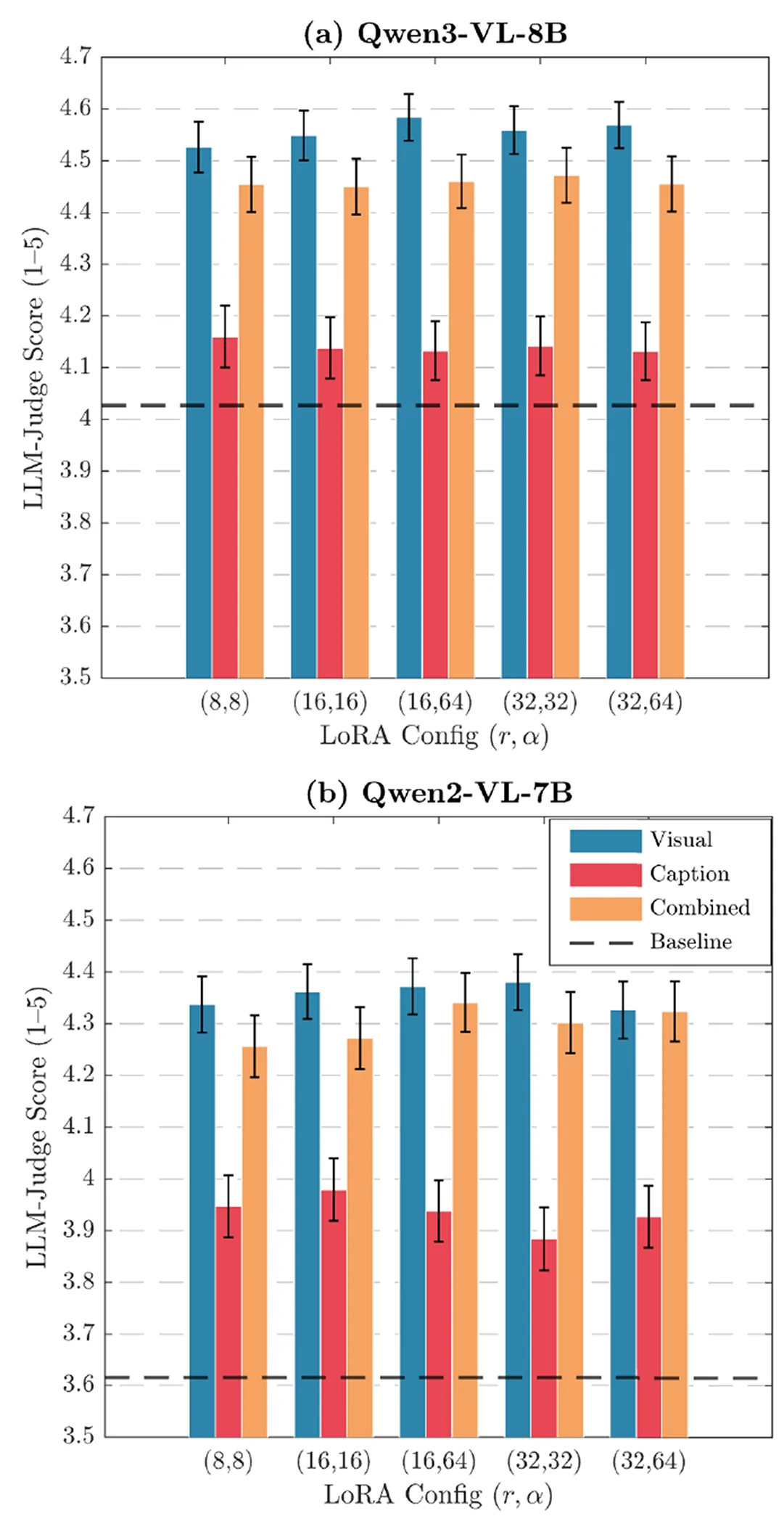
Ablation study on training data composition and LoRA configuration. LLM-as-Judge consensus scores (1–5) across five LoRA configurations and three training modes for (a) Qwen3-VL-8B and (b) Qwen2-VL-7B. The dashed line indicates the untuned baseline. Error bars denote 95% confidence intervals (*n*=1,201).

Figure 2 presents an ablation study examining the effects of training data composition and LoRA configuration. By LLM-judge consensus, visual-grounded training outperforms caption-based and combined modes on the 7B/8B backbones, supporting the notion that spatial and procedural visual cues aid TC3-specific reasoning. LoRA configuration has a comparatively modest, stable impact across all five settings, indicating that training-data composition contributes more substantially to adaptation quality than rank selection.

#### 1) LLM-JUDGE VERSUS REFERENCE-BASED METRICS: A RANKING INVERSION

The mode ranking is metric-dependent. While the LLM judges rank visual-grounded highest, the reference-based metrics invert this ordering. At the headline configuration, combined training achieves higher BERTScore-F1 (0.910 vs. 0.900) and ROUGE-L (0.358 vs. 0.300), with visual-grounded ranking lowest. This pattern is consistent across all five configurations and both the 7B and 8B backbones. On the 2B backbone, combined training also performs best on both families of metrics. We interpret this as a genuine measurement finding rather than noise. Visual-grounded training maximizes the qualities rewarded by the LLM judges, namely completeness and actionability of field-executable guidance, whereas combined training maximizes lexical and semantic overlap with the reference answers. Because the same model families that generated our training Q&A also serve as the LLM judges, we treat the reference-based metrics as a judge-independent cross-check and report both signals rather than relying solely on the judge ranking. We therefore characterize visual-grounded supervision as the configuration that best supports clinically actionable responses, while recognizing that combined supervision is stronger under reference-based metrics, especially on smaller backbones.

#### 2) PER-AXIS BREAKDOWN

Disaggregating the judge scores by rubric axis (Table 3) shows the gains concentrate on the axes tied to executable field care. For the headline Qwen3-VL-8B configuration, fine-tuning raises Completeness from 3.77 to 4.53 (+0.76) and Actionability from 4.13 to 4.67 (+0.54), larger than the gains on medical accuracy (4.01→4.49), protocol adherence (4.10→4.59), and safety (4.13→4.63); the same pattern recurs on the 7B and 2B backbones. This is consistent with visual-grounded supervision enhancing responses with procedural, field-executable detail that extends beyond factual accuracy.

To qualitatively assess the clinical impact of domain adaptation, we conducted a clinician evaluation in which two clinicians compared baseline and fine-tuned model responses on a randomly sampled subset of 40 test questions. We selected Qwen2-VL-7B as the comparison pair, evaluating its baseline against the visual-grounded fine-tuned variant. The Qwen3-VL-8B baseline was not used for this comparison, as its responses exhibited a tendency toward verbosity that could potentially compromise evaluator blinding and introduce response bias. Evaluators were presented with paired responses in randomized order and asked to select the clinically superior answer or indicate equivalence.

**FIGURE 3.**
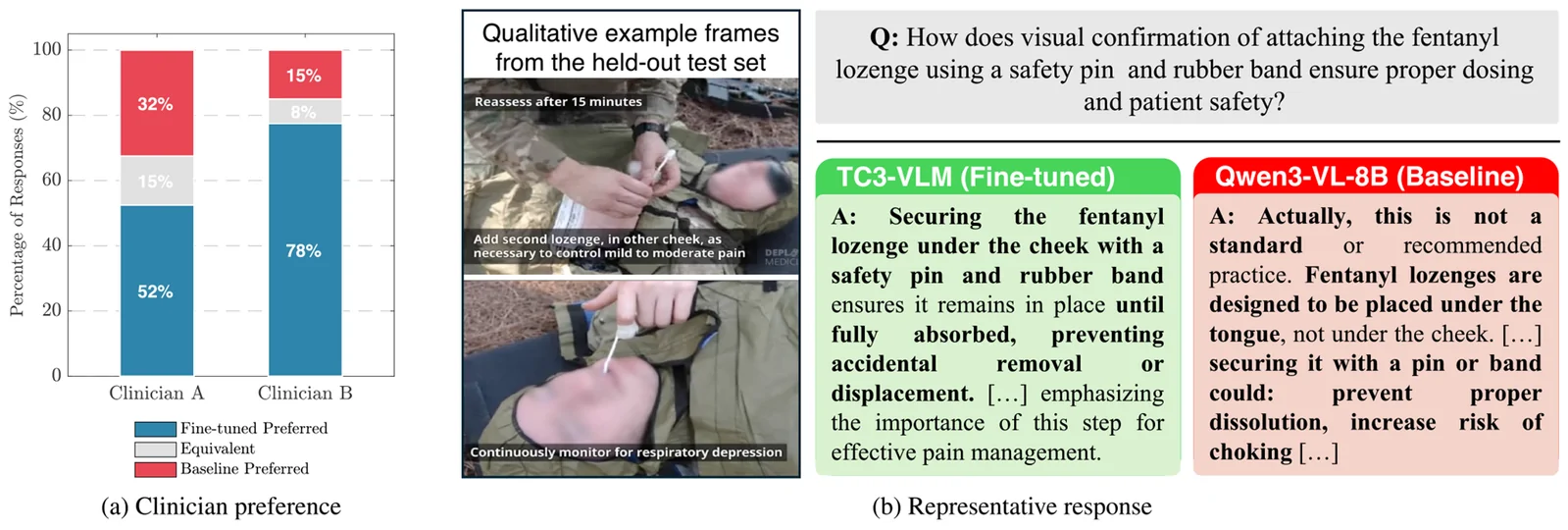
Qualitative analysis. (a) Blinded clinician pairwise preference between the fine-tuned TC3-VLM (Qwen2-VL-7B, visual-grounded) and its untuned baseline over 40 randomly sampled held-out test questions; both raters prefer the fine-tuned response in the majority of cases. (b) A representative held-out case complementing the per-category excerpts in Table 5: for a buccal (under-the-cheek) fentanyl lozenge—a valid TC3 analgesia procedure—the untuned baseline over-refuses (claiming lozenges belong under the tongue, citing a choking risk) while the fine-tuned TC3-VLM correctly explains the procedure.

As summarized in Figure 3(a), both clinicians preferred fine-tuned responses in the majority of cases (Clinician A: 52%, Wilson 95% CI [37, 67], Clinician B: 78%, [62, 88]), whereas the baseline was preferred in 32% and 15% of cases, respectively. Excluding equivalent responses, inter-rater agreement yielded a percent agreement of 77.4% and Cohen’s *κ* = 0.45, indicating moderate reliability [50]. When the decisive (non-equivalent) judgments from both raters are pooled, the fine-tuned responses are preferred in 73% of cases (Wilson 95% CI [62, 82]). Although the per-rater sample size is limited, the pooled preference remains significant under an exact binomial test (*p* < 0.001). These findings suggest that domain-specific fine-tuning enhances alignment with TC3 treatment protocols that may not be consistently captured by general-purpose models.

### D. ERROR AND SAFETY ANALYSIS

In addition to aggregate proxy metrics (e.g., BERTScore and LLM-Judge), we performed a TC3-grounded error analysis on a stratified random sample of held-out items to examine clinically relevant errors. A vision-capable LLM (Claude Opus 4.8, Anthropic) was shown the same multi-frame video montage provided to the VLM, together with the question, reference answer, and both model outputs. The model evaluated each baseline answer against the visual evidence and TC3 doctrine, classified erroneous responses into five predefined categories (Table 6), and determined whether the fine-tuned model fully, partially, or failed to correct the error. Doctrinal correctness was anchored to the CoTCCC TCCC Guidelines [1] and U.S. Army casualty-care doctrine (ATP 4-02.11) [51]; all identified errors were independently verified against these references and the underlying video frames. To minimize self-evaluation bias, the screening model was independent of both the models used to construct the dataset and the models under evaluation. As an automated LLM-based assessment, this analysis is intended to characterize failure modes.

Of the 149 sampled baseline answers, 41 (28%) contained a TC3 doctrinal error (Table 6). The most common failure modes were over-refusal and hallucination (12 cases each). Over-refusal occurred when the model rejected questions directly supported by the visual evidence, whereas hallucinations introduced procedures or observations absent from the frames. Additional errors included adjacent interpretation (7), lack of specificity (6), and civilian-default conflicts (4), in which civilian or hospital standards were substituted for TC3 doctrine.

Fine-tuning fully corrected 20 of the 41 errors and partially corrected 7 more, addressing 66% of identified failures. This suggests that domain adaptation improves more than lexical similarity or response style: it reduces clinically meaningful, frame-grounded doctrinal errors. Representative examples are shown in Table 5. Figure 3(b) illustrates a representative over-refusal case in which the baseline incorrectly rejects a valid TC3 procedure—a buccal fentanyl lozenge for analgesia—while TC3-VLM correctly explains the technique based on the same visual evidence.

## V. DISCUSSION

This study demonstrates that domain-specific fine-tuning can consistently surpass larger general-purpose models. Notably, despite being an earlier-generation and substantially smaller model, the fine-tuned Qwen2-VL-7B achieves performance comparable to the Qwen2.5-VL-72B baseline. This finding suggests that in highly specialized domains such as combat medicine, targeted adaptation can be more effective than scaling model size alone. Such efficiency is relevant for resource-constrained settings; we emphasize, however, that the present results establish improved performance on proxy and preliminary clinician measures, not validated readiness for autonomous field decision support.

Visual-grounded training also yields the strongest judgerated performance among the three training modes, consistent with the importance of spatial and procedural visual cues in TC3 reasoning, where equipment recognition and anatomic landmarks directly inform clinical actions. We further observe a consistent domain-knowledge conflict in baseline models, which default to civilian medical standards in combat settings. This misalignment reflects a limitation of general biomedical pretraining and motivates explicit domain-specific adaptation for safety-critical applications.

These findings also highlight the evolving role of AI in combat casualty care. Beyond the physiological prediction and decision-support systems that have dominated prior work, recent efforts increasingly focus on autonomous and generative assistance at the point of injury. For example, the DARPA Triage Challenge aims to advance automated casualty assessment [52], while passive multimodal documentation platforms capture injury scenes to create datasets for downstream learning [53]. In parallel, general-purpose large models are being applied to acute triage, although their performance remains limited without domain adaptation, leading to over-triage and misclassification of high-acuity cases [34]. TC3-VLM complements these directions by enabling open-ended visual reasoning and protocol-oriented instruction directly from casualty scenes. Its scene-level understanding could provide contextual input to autonomous triage systems, while multimodal records collected by passive documentation platforms could serve as valuable training data for adapting TC3-VLM to authentic point-of-injury conditions.

This study has several limitations. Although clinicians were involved throughout Q&A generation and prompt review, the final quantitative evaluation relied on two clinicians assessing 40 sampled questions, limiting the breadth of expert validation. Accordingly, we frame this study as a pilot evaluating whether domain-specific adaptation instills TC3-aligned knowledge. The primary evaluation is automatic over 1,201 held-out items and is complemented by a 40-question clinician study. In addition, there is currently no standardized benchmark for TC3-specific vision–language question answering, making comparisons across studies and evaluation settings difficult. A systematic expert audit of a representative sample of the generated training pairs also remains an important direction for strengthening confidence in the dataset. Generalization remains an open question, as our corpus is mainly derived from publicly available instructional and training videos rather than authentic point-of-injury imagery. Although we included field footage of varying quality to improve data diversity, the resulting corpus remains dominated by clean, well-framed instructional videos. As a result, the recognition and grounding learned by TC3-VLM may not fully generalize to the occlusion, motion, and degraded imagery encountered in real point-of-injury settings. Finally, both the training Q&A and the error-analysis annotations incorporate LLM-assisted labeling. Although these procedures enabled scalable dataset construction and analysis, they may introduce systematic biases and cannot replace clinician-adjudicated ground truth.

Future work should focus on both translational deployment and methodological validation. From a systems perspective, enabling real-time interaction in resource-constrained field environments will require advances in efficient inference, low-latency response generation, and edge deployment. From a modeling perspective, our results suggest that visual-grounded training is particularly effective for spatial and procedural reasoning, whereas caption-based supervision may provide complementary benefits for protocol-oriented knowledge. Integrating structured TC3 guidelines directly into multimodal training, or combining TC3-VLM with retrieval-augmented generation linked to authoritative medical references and operational protocols, may further improve factual grounding, procedural consistency, and domain alignment. Future evaluation should also extend to authentic operational imagery, larger-scale clinician-adjudicated benchmarks, and more comprehensive expert review. Finally, although this study focused on the Qwen-VL family, determining whether similar domain-adaptation gains transfer to other VLM architectures remains an important direction.

## VI. CONCLUSION

We presented TC3-VLM, the first vision–language model specifically fine-tuned for Tactical Combat Casualty Care. Our results show that domain-specific fine-tuning substantially improves performance over general-purpose baselines, highlighting the importance of explicit domain adaptation in combat casualty care. We further demonstrate that visual-grounded training is essential for capturing the spatial and procedural reasoning required in combat medicine. By improving efficiency without relying on large model scale, this work supports the development of lightweight training and reference aids for combat casualty care and establishes a pathway toward future field-deployable multimodal decision-support systems, contingent on rigorous clinician validation and real-world evaluation.

## Figures and Tables

**TABLE 1.** Positioning of TC3-VLM relative to representative medical VLM, medical-QA, and surgical/medical-video work.

Work	Domain	Source data	Modality	Supervision
LLaVA-Med [22]	general biomedical	PMC figures	image	model-generated instruction tuning
PMC-VQA [23]	general biomedical	PMC figures	image	generative VQA
VQA-RAD [24]	radiology	radiology images	image	clinician-authored QA
PathVQA [25]	pathology	textbook captions	image	caption-derived QA
MedVidQA [26]	general medical	instructional video	video	human-annotated QA
Thapa et al. [27]	general biomedical	public educational video	video	video instruction tuning
LLaVA-Surg [28]	surgery (in-OR)	public surgical video	video	LLM-generated QA
Surgical-VQA [29]	surgery (in-OR)	curated surgical video	video	closed-set VQA
Nemeth et al. [10], [11]	TC3 / prehospital	vital-sign time series	tabular	supervised prediction
Med-PaLM 2 [18]	general medical	medical exams	text	instruction tuning / RLHF
**TC3-VLM (ours)**	**TC3 combat care**	**public TC3 video**	**video**	**visual-primary + caption QA + LoRA**

**TABLE 2.** Representative raw transcripts and their refined captions across TC3 topics. Refinement removes verbal fillers and self-introductions, corrects speech-recognition errors, and normalizes terminology while preserving content. Excerpts trimmed with “…”.

Topic	Raw transcript (subtitles/ASR)	Refined caption
Medication (IO)	Hello, I’m Adam, an active duty Air Force medic. I’m going to be teaching you how to administer medications via the *inter-Oscius* route. This is done in the tactical field care setting…	Adam, an active duty Air Force medic, instructs on administering medications via the *intraosseous (IO)* route in tactical field care… when a casualty with established IO access requires IV medications.
Airway	Airway Management. For a conscious casualty with no airway problems, no airway interventions are needed… Place the casualty in the recovery position. Use the chin lift or jaw thrust maneuver… or *extraglotic* airway…	For a conscious casualty without airway issues, no intervention is required… place them in the recovery position and apply either a chin lift, jaw thrust, nasal pharyngeal airway (NPA), or an *extraglottic* airway (EGA).
Hemorrhage/shock	Shock is inadequate blood flow to the body tissues… The normal adult blood volume is 5 liters. 500 ml of blood loss is when you donate a unit… After 1000 ml… a slightly elevated heart rate.	Shock is characterized by inadequate blood flow to body tissues, often from severe blood loss on the battlefield… normal adult blood volume is approximately 5 liters, a donation equates to about 500 ml… after 1000 ml, symptoms might include a slightly elevated heart rate.
Evacuation	…make sure it’s ran to the inside. And *um, it’s good. All right.* Prepare the head portion of the sked litter for a hoist operation. There’s now *gonna* work on the head…	Prepare the head section of the SKED litter for a hoist operation by removing slack and guiding the head portion over the casualty… ensuring the curved part does not touch the casualty’s head.

**TABLE 3.** Main results across model scales. Fine-tuned models use visual-grounded training with (*r, α*) = (16, 64) LoRA configuration. Qwen2.5-VL-72B is included as a large-scale baseline reference. All improvements are statistically significant (paired *t*-test, *p* < 0.001).

Model	Setting	LLM-Judge						BERTScore
		M	P	C	A	S	Overall	
**TC3-VLM** (Qwen3-VL-8B)	Fine-tuned	**4.49**	**4.59**	**4.53**	**4.67**	**4.63**	**4.584**	**0.900**
	Baseline	4.01	4.10	3.77	4.13	4.13	4.027	0.857
Qwen2-VL-7B	Fine-tuned	4.27	4.41	4.26	4.50	4.42	4.372	0.900
	Baseline	3.48	3.78	3.33	3.82	3.68	3.615	0.892
Qwen2-VL-2B	Fine-tuned	3.54	3.72	3.46	3.83	3.72	3.655	0.893
	Baseline	3.12	3.36	3.03	3.38	3.30	3.238	0.888
Qwen2.5-VL-72B	Baseline	4.37	4.44	4.08	4.45	4.46	4.360	0.876

M: Med. Acc., P: Protocol Adh., C: Completeness, A: Actionability, S: Safety

**TABLE 4.** Inter-judge consistency between Llama-3.1-405B and Qwen2.5-72B across model scales and settings. Agr. (±1): fraction of items on which the two judges agree within one point; MAE: mean absolute error between their scores.

Model	Setting	Pearson *r*	Spearman *ρ*	Agr. (±1)	MAE
**TC3-VLM** (Qwen3-VL-8B)	Fine-tuned	0.729	0.728	91.5%	0.306
	Baseline	0.576	0.481	75.5%	0.770
Qwen2-VL-7B	Fine-tuned	0.762	0.747	88.0%	0.407
	Baseline	0.777	0.804	78.6%	0.636
Qwen2-VL-2B	Fine-tuned	0.815	0.813	81.0%	0.608
	Baseline	0.814	0.830	80.5%	0.617
Qwen2.5-VL-72B	Baseline	0.769	0.640	92.3%	0.403

**TABLE 5.** Representative baseline errors verified against the held-out video frames, each shown with the fine-tuned model’s response to the same item (excerpts trimmed with “…”; clinically decisive spans kept verbatim).

Category	Question	Baseline	Fine-tuned
**Over-refusal**	During the chest-seal “burping” step shown, what visual cue indicates the seal must be repositioned?	*…there is no such thing as “burping” a wound… this term is not medically accurate or used in trauma care…*	…if the seal is improperly placed you may see continued air leakage… repositioning ensures a tight seal, preventing further air entry…
**Adjacent interpretation**	How does the tourniquet’s position relative to the bony prominences affect its efficacy?	*…positioned directly over the bony prominences… bony prominences provide a stable anchor point…*	…compresses the artery against the bone… visually confirmed by the absence of a distal pulse…
**Civilian-default**	What is the responder’s next step after needle decompression and taping the chest?	*…the next step is to transport the patient to a hospital for definitive care…*	…assess the patient’s breathing and circulation, checking for further respiratory distress or shock… before moving on to other injuries…
**Lack of specificity**	Why check the patient’s response to commands during the lower-extremity examination?	*…a component of neurological and musculoskeletal assessment… assessment of motor function (“raise your leg,” “resist my push”)…*	…helps assess the patient’s level of consciousness and neurological function… whether the patient is alert and oriented…

**TABLE 6.** Baseline error taxonomy and the effect of fine-tuning, on a stratified held-out sample (149 items; 108 acceptable, 41 errors; TC3-prompted screening coding).

Baseline error type	Errors	Fixed^a^	Partial^a^
Over-refusal	12	10	2
Hallucination	12	4	1
Adjacent interpretation	7	3	3
Lack of specificity	6	2	1
Civilian-default	4	1	0

**Total**	**41**	**20**	**7**

a Number of that row’s errors that fine-tuning fully corrects (*Fixed*) or partially corrects (*Partial*); any remainder is left uncorrected.
